# Supplementary material for: Bio-inspired reversible underwater adhesive
Source: Nat Commun. 2017 Dec 20;8:2218. doi: 10.1038/s41467-017-02387-2 (PMC5738439; doi:10.1038/s41467-017-02387-2)
Supplement: Supplementary file 1 — Supplementary Information [file 41467_2017_2387_MOESM1_ESM.pdf]

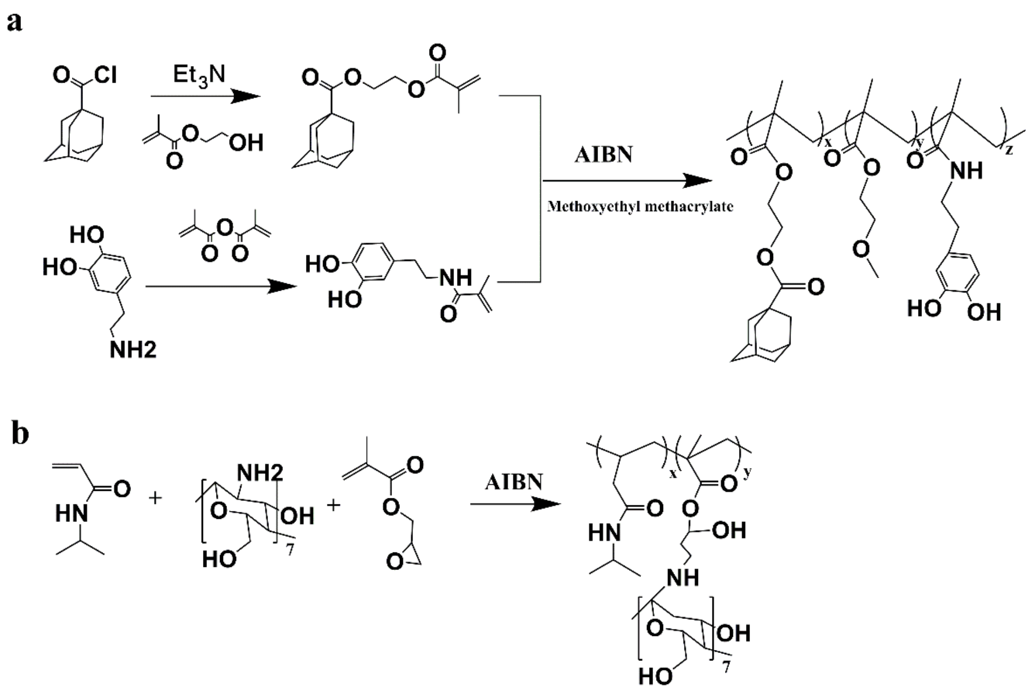

Supplementary Figure 1 | Synthetic scheme of the guest and host copolymers. **(a)** Guest copolymer pDOPA-AD-MEA is synthesized via free radical polymerization of AD monomer, DOPA monomer, and MEA. (**b**) Preparation of host copolymer pNIPAM-CD by the copolymerization of NIPAM and CD.

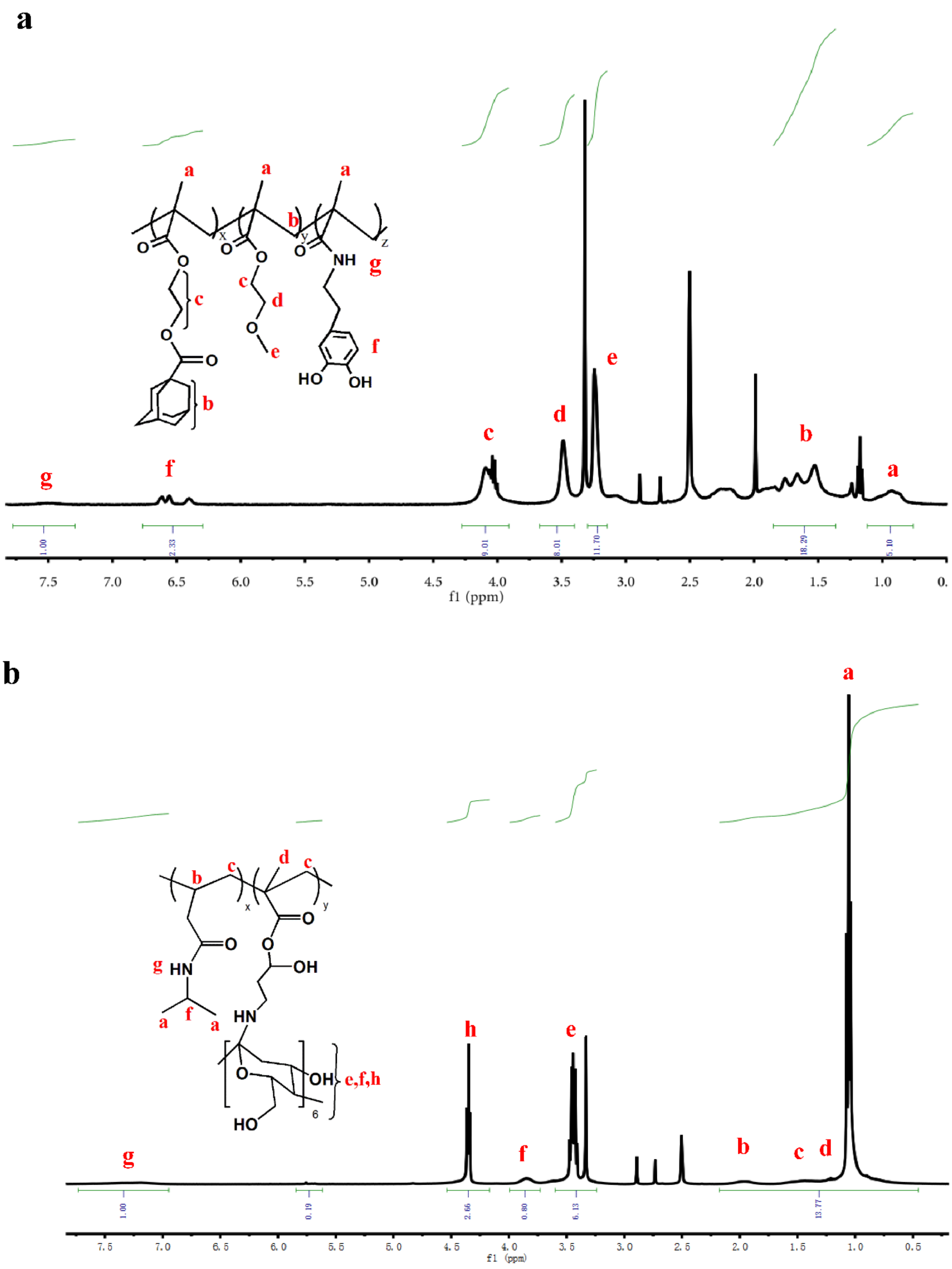

Supplementary Figure 2 | The ^{1}H NMR spectrum of the guest copolymer pDOPA-AD-MEA (a) and host copolymer pNIPAM-CD (b) in DMSO. The characteristic NMR peaks corresponding to pDOPA-AD-MEA and pNIPAM-CD are clearly labeled.

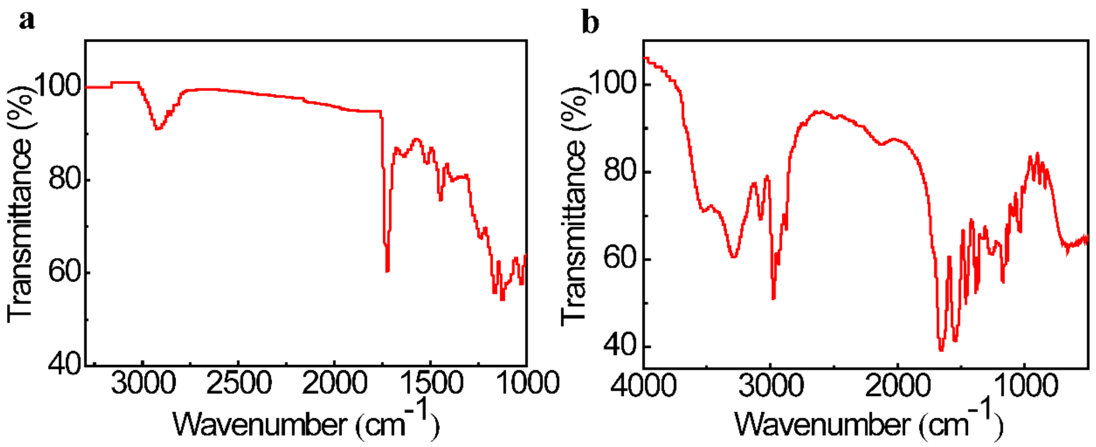

Supplementary Figure 3 | FTIR spectra of the guest copolymer pDOPA-AD-MEA (a) and host copolymer pNIPAM-CD (b). (**a**) The characteristic peaks corresponding to the phenolic hydroxyl group (3430 cm^{-1}), ester acyl group (1730 cm^{-1}), and benzene group (1640) are clearly illustrated, suggesting the successful synthesis of the pDOPA-AD-MEA. (**b**) The characteristic peaks at 3290 cm^{-1}, 2970 cm^{-1}, 1660 cm^{-1}, and 1560 cm^{-1} are assigned to the cyclodextrin hydroxyl group, methyl-methylene group, carbonyl group, and imino group, respectively, demonstrating the successful fabrication of the pNIPAM-CD.

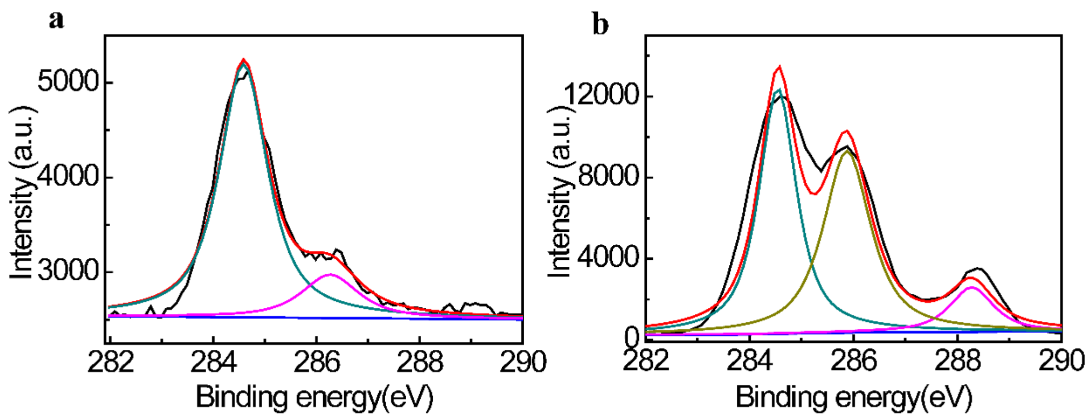

Supplementary Figure 4 | High-resolution C1s XPS spectrum analysis of host copolymer and the adhesive coating. (**a**) The characteristic peaks at 284.8 eV and 286.3 eV observed in the XPS spectrum are assigned to the C1s absorption of the ether group in pDOPA-AD-MEA. (**b**) After assembly of the host copolymer, an additional C1s absorption peak at 288.3 eV emerges, which corresponds to the carbonyl amide group in pNIPAM-CD.

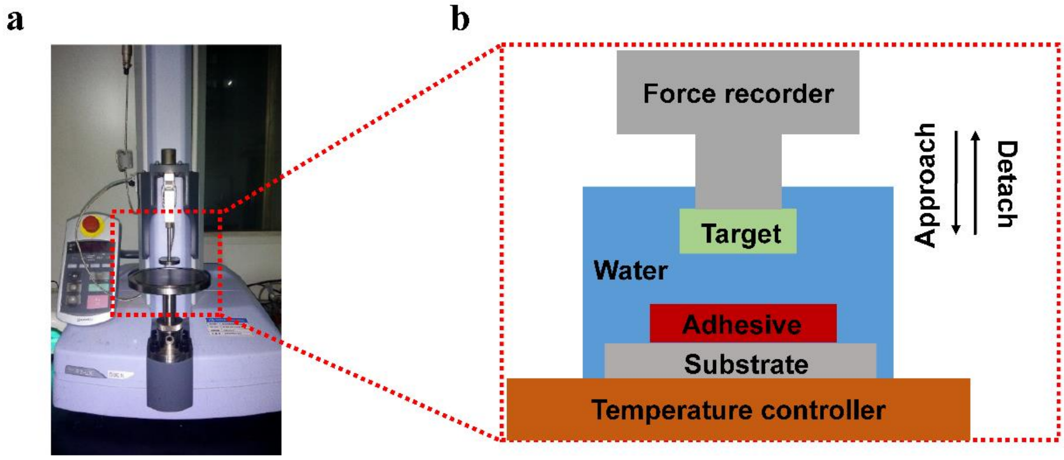

Supplementary Figure 5 | Optical image of the UTM setup for the measurement of the interfacial adhesion. (**a**) Optical photograph of UTM. (**b**) Schematic drawing showing the detailed set-up to characterize the interfacial adhesion.

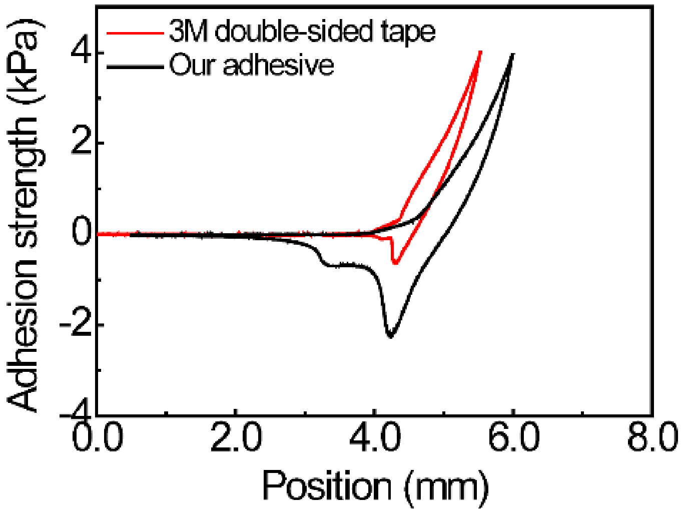

Supplementary Figure 6 | Comparison of adhesion performances of adhesive coating and 3M double-sided tape. In the wet environment, the adhesion strength of our adhesive is 5 times stronger than that of commercially available 3M double-sided tape.

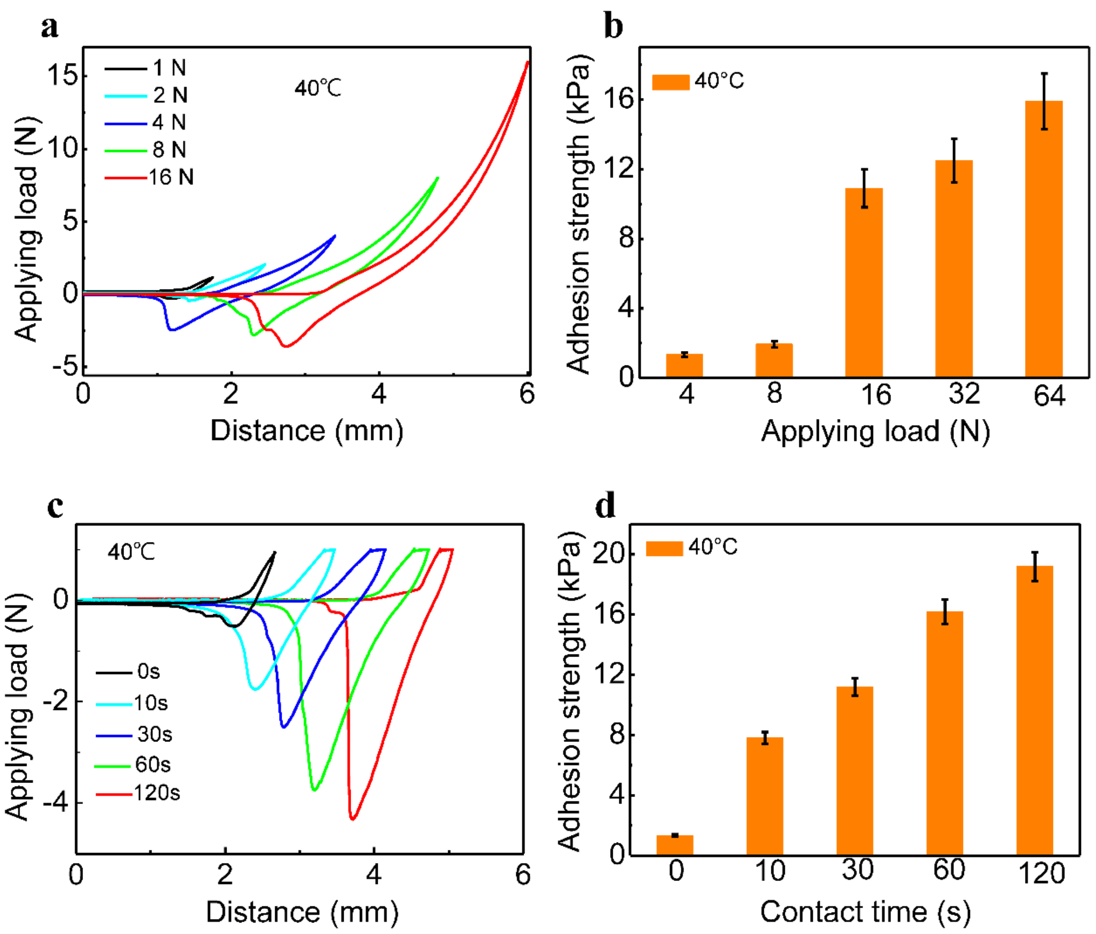

Supplementary Figure 7 | Tailoring underwater adhesion by tuning pre-applying load and contact time. (**a, b**) The variation of the adhesion properties under different applying loads. Here the contact time is maintained at 0 s and the temperature is 40 °C. (**c, d**) The variation of the adhesion properties under different contact times. Here the applying load is maintained at 1 N and the temperature is 40 °C. For an applying load of 1 N and contact time of 120 s, the adhesion strength is increased to 18 kPa.

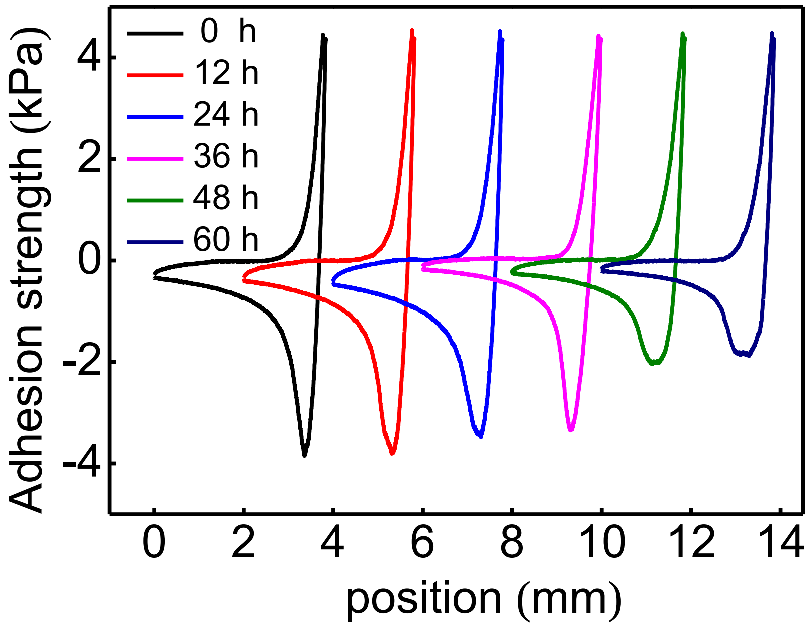

Supplementary Figure 8 | Time-dependent variation of the adhesion strength at 40ºC. The adhesion strength of the adhesive coating is quite stable in the first 36 h and up to a continuous operation of 48 h, there is a marked drop in the adhesion strength.

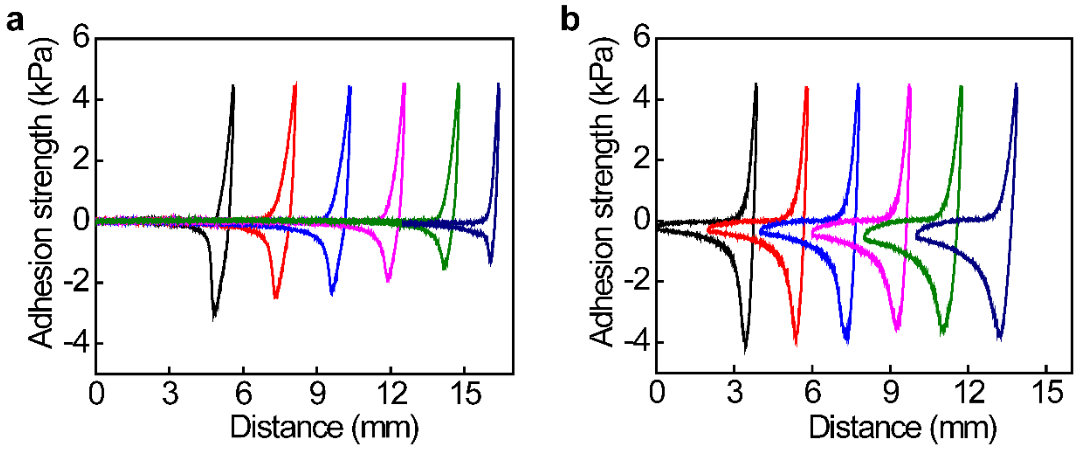

Supplementary Figure 9 | Comparison of wet adhesion strength between control sample (AD-MEA-coated substrate) and AD-MEA-DOPA coated substrate at 40°C. (**a**) For the control sample, the adhesion strength exhibits a significant decay during successive tests. The curves in black, red, blue, pink, green and navy blue correspond to the tests conducted on 1st, 11th, 21st, 31st, 41st, and 51st cycle, respectively. (**b**) For the wet adhesive made of AD-MEA-DOPA, there is no marked decay in the adhesion strength even after 50 cycles of tests.

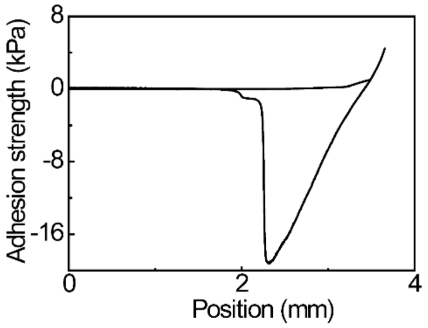

Supplementary Figure 10| Adhesion strength of the adhesive coating in the air. The adhesion strength of the adhesive coating at 25°C and 40°C in air is ~20 kPa, demonstrating that the thermo-reversible adhesion cannot be achieved in dry condition.

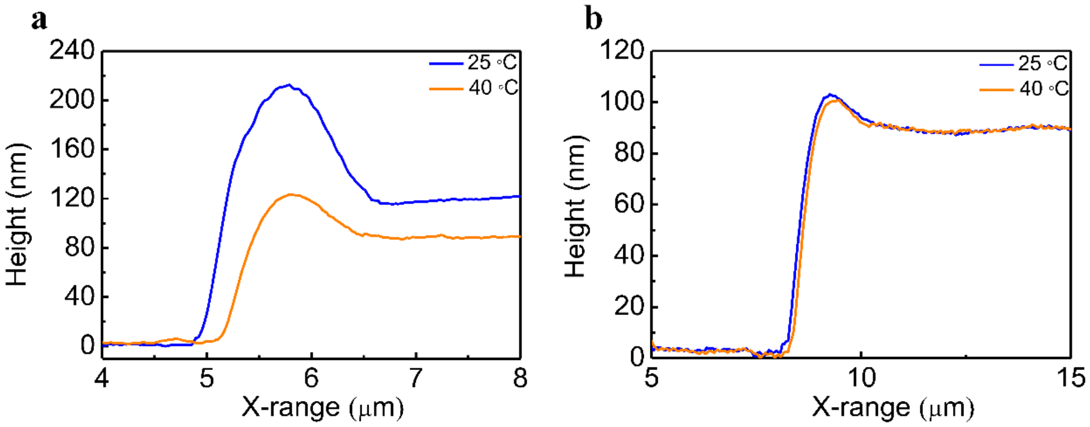

Supplementary Figure 11 | Thickness measurement of the adhesive coating in wet and dry state. (a) In the wet environment, the thickness of the adhesive coating at 25 ^{o}C is 115 nm, which is larger than that at 40 ^{o}C (90 nm). (b) In the dry environment, the thickness of the adhesive coating at 25 ^{o}C is 80 nm, which is almost the same as that at 40 ^{o}C.

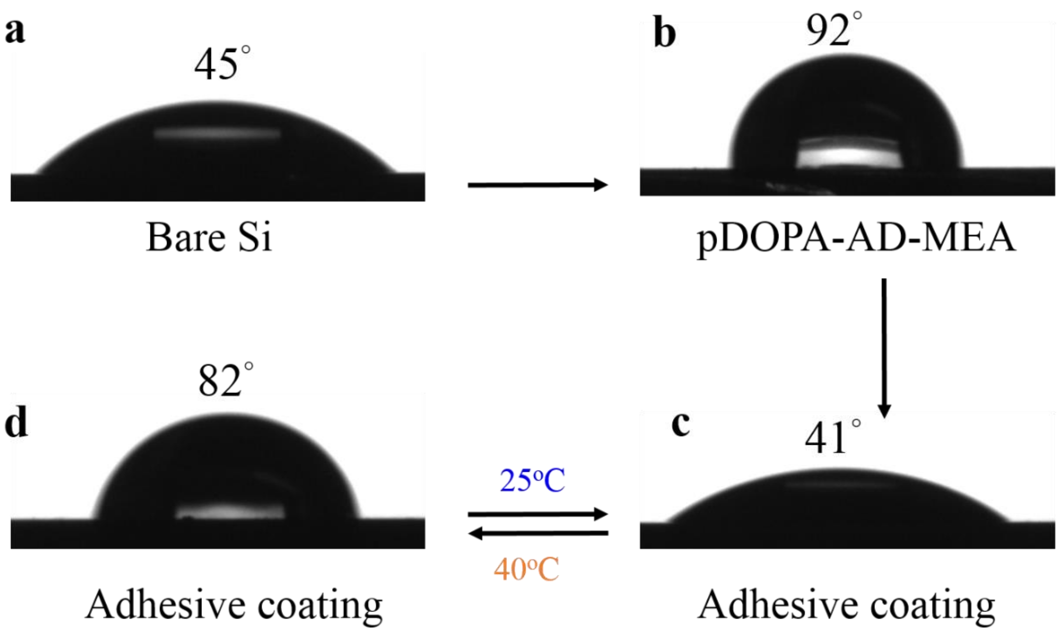

Supplementary Figure 12 | Contact angle (CA) measurement. (**a**) A thoroughly cleaned silicon substrate exhibits a hydrophilic property with a water CA of 45°. (**b**) The water CA is increased to 92° after the deposition of the pDOPA-AD-MEA on the silicon substrate due to the existence of hydrophobic MEA monomer in the guest copolymer. (**c**) After the self-assembly of pNIPAM-CD, the water CA at 25°C is reduced to 41°. (**d**) At 40°C, the adhesive surface becomes hydrophobic with a CA of 82° due to the collapse of pNIPAM side-chains.

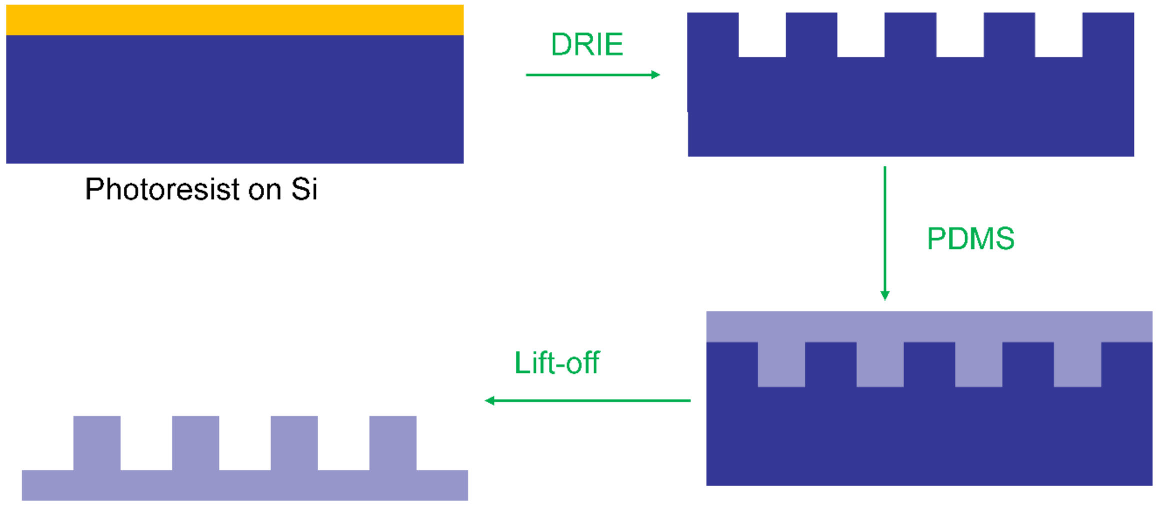

Supplementary Figure 13 | Schematic diagram showing the detailed process to fabricate PDMS pillar arrays. A silicon template with micro-hole arrays was first fabricated using photolithography and dry etching. Then PDMS was cast onto the as-fabricated template, followed by curing and lift-off.

Supplementary Table 1. Element content analysis of bare silicon, pDOPA-AD-MEA, and adhesive coating

**Element content (%)**	**C**	**O**	**N**
**Bare Si**	31.41	68.59	0
**pDOPA-AD-MEA**	72.63	27.04	0.89
**Adhesive coating**	71.63	24.04	3.89
