# Supplementary material for: Bio-inspired reversible underwater adhesive
Source: Nat Commun. 2017 Dec 20;8:2218. doi: 10.1038/s41467-017-02387-2 (PMC5738439; doi:10.1038/s41467-017-02387-2)
Supplement: Supplementary file 3 — Description of Additional Supplementary Files [file 41467_2017_2387_MOESM3_ESM.pdf]

Description of Additional Supplementary Files

File Name: Supplementary Movie 1
Description: Underwater place-and-pick experiment at 40 ^{o}C. The as-prepared adhesive coating was first decorated on a heat conducting aluminum sheet whose temperature was controlled by a hot plate. At 40 ^{o}C, we approached the adhesive to a copper block (200 g). After about 10 s contact time, the copper block was able to be picked up by the adhesive coating.

File Name: Supplementary Movie 2
Description: Underwater place-and-pick experiment at 25 ^{o}C. When the local temperature was changed to 25 ^{o}C, the specimen cannot be picked up by the adhesive coating due to the supramolecular screening effect.
